# Breast Self-Examination Knowledge and its Determinants among Female Students at Addis Ababa University, Ethiopia: An Institution-Based Cross-Sectional Study

**DOI:** 10.1155/2022/2870419

**Published:** 2022-05-30

**Authors:** Mikiyas Amare Getu, Mesfin Abebe, Kenean Getaneh Tlaye, Abel Tibebu Goshu

**Affiliations:** ^1^Woldia University, Faculty of Health Sciences, Department of Nursing, Ethiopia; ^2^Addis Ababa University, College of Health Sciences, School of Nursing and Midwifery, Ethiopia; ^3^Haramaya University, College of Health and Medical Sciences, School of Nursing and Midwifery, Ethiopia

## Abstract

**Introduction:**

Breast self-examination is a noninvasive, low-cost screening method for breast cancer detection. A thorough awareness of breast self-examination enables the early detection of breast abnormalities and dramatically lowers breast cancer complications and mortality. The purpose of this study was to investigate the level of knowledge about breast self-examination and its associated factors among female students at Addis Ababa University, Ethiopia.

**Methods:**

An institution-based cross-sectional study design was employed. The final calculated sample size was 407, and participants were recruited using a proportionate stratified random sampling approach. For data entry and coding, EPI Data 3.1 statistical software was utilized, and for data analysis, SPSS version 18 was employed. The data was described using descriptive analysis. Bivariate and multivariate logistic regression analyses were performed to determine the strength of the association between the predictor and the outcome variables. A 95% confidence interval and a *p*-value of less than 0.05 were used to declare statistical significance.

**Results:**

The findings of this study revealed that 49.9% of respondents possessed good breast self-examination knowledge. Previously, urban residents were about two times more likely to have good knowledge of BSE than their rural counterparts (AOR =2.16, 95% CI (1.18–39.91), p =0.011). The odds of having good BSE knowledge were about three times more likely among those who had a good attitude than those who had a poor attitude (AOR =3.17, 95% CI (2.02–4.74), p <0.001). Those who knew someone with a diagnosis of breast cancer were almost three times more likely to have good knowledge than those who did not know (AOR =2.95, 95% CI (1.77-4.91), p <0.001).

**Conclusion:**

According to the findings of this survey, less than half of the students who participated had good knowledge of breast self-examination. This justifies raising awareness about breast self-examination among female students.

## 1. Introduction

Breast cancer (BC) is the most common malignancy in women worldwide, accounting for 2,261,419 new cases and 684,996 deaths [1]. Furthermore, it is the leading cause of cancer death in the vast majority of the world's low-income countries [1, 2]. While industrialized countries have a higher incidence of BC, less developed countries have a higher mortality rate. This is due to a lack of early detection and management services and a limited awareness of early cancer signs and symptoms among the public and healthcare providers [1].

In Africa, BC primarily affects younger or premenopausal women [2]. It is most common in southern Africa and the least common in middle Africa [2–4]. However, breast and cervical cancer are among the most common types of cancer in most east African countries, including Ethiopia. The mortality rate is comparable to that of other regions of the continent and significantly higher than that of the majority of developed countries [1, 3]. The age-standardized BC mortality rates in East Africa and Ethiopia are 17.9 per 100,000 women and 22.9 per 100,000 women, respectively [1, 5].

There are opportunities to reduce cancer-related suffering and death at all stages of the cancer control spectrum, from prevention to early detection, treatment, and palliative care. However, Breast Self-Examination (BSE) stands out as a simple, low-cost, quick, and noninvasive early detection method for BC among African women [6–12]. BSE assists women in detecting changes in their breasts early and familiarizes them with their appearance and feel. It also encourages them to seek medical attention, such as clinical breast examinations (CBE) and mammography [11, 13].

Adequate use of effective preventive measures, such as BSE, is closely related to disease awareness and the how-to and benefits of self-examination techniques [14]. A thorough understanding of BSE allows early detection of breast abnormalities and significantly reduces BC-related complications and fatalities. Studies that assessed BSE knowledge among young college students paint a contrasting picture. The knowledge level is 18% in India, 38.9% in Malaysia, 68.5% in the UAE, 55.3% in Nigeria, and 73.5% in Cameroon [15–19]. Studies from Adama University and Gondar University in Ethiopia found that only 8.7% and 27.6% of students, respectively, had good knowledge of BSE [11, 20].

Most girls in their twenties enroll in state-run higher health education institutes located far from their birthplace in Ethiopia. It is time they gained their autonomy in choosing free and informed reproductive choices [21]. Identifying knowledge gaps in reproductive health and empowering these youngsters is critical at this point. To this end, it is necessary to determine the level of awareness about BSE among these age groups. However, to the best of the investigators' knowledge, scant evidence is available to derive interventional strategies. As a result, this study aimed to assess the BSE knowledge of students not majoring in health sciences at Addis Ababa University (AAU).

## 2. Methods

The STROBE (Strengthening the Reporting of Observational Studies in Epidemiology) standards were utilized to report this cross-sectional study and make the presentation of the findings adequate, transparent, and complete. The checklist includes recommendations for the following sections of the report: title and abstract; introduction; methods; results; discussion; and other information. (Supplementary file).

### 2.1. Study Setting and Period

The study was carried out at Addis Ababa University's College of Business and Economics in Ethiopia from February 1–30, 2016. The College of Business and Economics (CoBE) was formed in November 1990 by the merger of the previous Faculty of Business and Economics and the School of Commerce (established in 1943). It is divided into four departments (Accounting and Finance, Economics, Management, and Public Administration and Development Management) and six programmes within the School of Commerce. AAU enrolled approximately 33,940 undergraduate students, 13,000 graduate students, and 1733 PH.D. students, for a total of 48,673 students. A total of 3221 students were pursuing their education at the CoBE during the study period. There were 940 female undergraduate students, 350 from the Faculty of Business and Economics and 590 from the School of Commerce [22].

### 2.2. Study Design

An institution-based analytical cross-sectional study was employed.

### 2.3. Study Participants

All female undergraduate students enrolled at Addis Ababa University were the source population, and all female undergraduate students attending their education in selected departments at Addis Ababa University were the study population. Regular female undergraduate students enrolled during the study period and aged 20 years or older were included. Students who were absent from the classroom for different reasons during the data collection period and those who were seriously ill and unable to communicate were excluded.

### 2.4. Study Variables

The outcome variable was BSE knowledge. Socio-demographic parameters (age, marital status, year of study, prior place of residence, average family income), personal history of benign breast abnormalities, family history of breast cancer, and attitude toward BSE were considered independent factors.

### 2.5. Sample Size and Sampling Technique

The sample size was determined using a single population proportion formula with the assumptions of a 95% confidence interval, a margin of error of 5%, and a *p*-value of 59.5% [23]. After adding a 10% non-response rate for an incomplete or lost questionnaire, the total sample size was 407. Students from the CoBE were chosen using a proportionate stratified random sampling technique. Six departments were considered strata, and each year of study (Year I–Year III) was a sub-strata. Each stratum received a proportional allocation, and participants were chosen randomly.

### 2.6. Data Collection Instrument

BSE knowledge was assessed using a structured questionnaire adapted from an intensive literature review [23–26]. The questionnaire was written in English, translated into Amharic, and then translated back to English for consistency checks. Five sections which assessed socio-demographic characteristics, family history of breast cancer, attitude towards BSE, and BSE knowledge were included in the questionnaire. The reliability (internal consistency) of the items was checked using Cronbach's alpha and was found to be greater than 0.70. Experts evaluated the tool's content validity, and all items were deemed relevant. Scores for the item-level content validity index (I-CVI) ranged from 0.83 to 1.

### 2.7. Measurement of Variables

BSE knowledge was assessed with 10 questions, with possible scores ranging from 0 to 10. Attitude towards BSE was measured using eight items (four negative attitude items and four positive attitude items) with five Likert scale options (strongly agree/agree/neutral/disagree/strongly disagree) [24, 25, 27, 28]. The possible scores for the attitude-related items ranged from 8 to 60.

### 2.8. Operational Definitions

Regular BSE - A woman who routinely performs BSE once a month.

Good knowledge – A score equal to or above the mean value of the knowledge items.

Poor knowledge – A score less than the mean value of the knowledge items.

Good attitude – A score equal to and more than the mean of attitude questions.

Poor attitude – A score less than the mean of attitude questions.

### 2.9. Data Quality Assurance

A comprehensive training focusing on the data collection tools, the data collection process, and research ethics was provided for data collectors and supervisors. The completeness, consistency, and appropriatness of filled questionnaires were checked daily by the data collectors, supervisors, and principal investigators. A pretest was performed on 5% of the sample size in a health care facility that was not included in the final study.

### 2.10. Data Processing and Analysis

For data entry and coding, EPI Data 3.1 statistical software was utilized, and for data analysis, SPSS version 18 was employed. The data was described using a descriptive analysis. To examine the relationship between predictor factors and the outcome variable, bivariate logistic regression was used. The factors that demonstrated a significant association with the outcome variable at a *p*-value of <0.2 were included in the multivariate logistic regression. The Hosmer-Lemeshow test was used to assess the model's fitness. The adjusted odds ratio with its 95 percent confidence interval and a *p*-value less than 0.05 were used to indicate statistical significance.

### 2.11. Ethical Considerations

The study was conducted after getting ethical clearance from the Research Ethical Committee of the School of Nursing and Midwifery at Addis Ababa University (Ref. no. 022/16/SNM). Permission was sought from the responsible authorities of selected colleges before data collection. Informed verbal consent was obtained from study participants.

## 3. Results

### 3.1. Socio-Demographic Characteristics

The research included 407 students, with a response rate of 100%. 347 (85.3%) respondents were aged 20–22 years, while 60 (14.7%) were 23 years or older. 342 (84%) of the research participants were from urban regions. 170 (41.8%) were second-year students, and most (61.4%) reported their families' income was more than 3500 Ethiopian Birr (approximately more than 161 USD). Around 44.7% of the study participants were Amhara, and 23.6% were Oromo (Table 1).

### 3.2. Personal and Family History

41 (10.1%) of the participating students reported a family history of BC, with 21 (51.2%) of them being their aunts. Of the total, 13 (3.2%) indicated that they had a personal history of benign breast problems, and 99 (24.3%) knew someone with BC (Table 2).

### 3.3. Breast Self-Examination Knowledge

Almost half (49.9%) of the survey participants had good BSE knowledge. Most (376, or 92.4%) of the students stated that they have ever heard of BSE. Just over half of the respondents (50.9%) affirmed that they had received information about BSE, of which most (64.2%) mentioned the mass media as their source of information. About 32% of respondents correctly identified that the proper age to begin BSE is after 19 years. About 229 (56.2%) of the respondents were familiar with CBE. Approximately 56% of participants reported that they did not know how often BSE should be performed, and 101 (24.8%) appropriately mentioned it should be done every month. Almost two-thirds of the participants (64.2%) did not know the right time to conduct BSE, and 60 (14.7%) replied with the correct answer after menstruation. Early detection of BC improves one's chances of survival, according to 69.3% of those who responded. Of the 376 respondents who have ever heard of BSE, 198 (52.6%) knew that BSE enables the detection of atypical changes in the size and form of the breasts. 152 (40.4%) did not know that BSE should be performed in front of a mirror (Table 3).

### 3.4. Attitude towards Breast Self-Examination

About 206 (50.6%) of the participants had a good attitude towards BSE. 73(17.9%) respondents agreed that doing BSE makes them feel absurd. Approximately two-thirds (63%) of respondents agreed or strongly agreed that they were motivated to perform BSE. Over half (54%) of respondents agreed or strongly agreed to discussing BSE with their friends. Only 5.9% of the participants strongly agreed that they would constantly look for information about BSE. An equal number of respondents (39.1%) disagreed and strongly disagreed that performing BSE is a waste of time. About 130 (31.9%) respondents strongly agreed that they hesitate to perform BSE as it is awkward; 260 (63.9%) agreed or strongly agreed that they avoid performing BSE due to concerns about developing breast cancer, and 65 (16%) strongly agreed or agreed that performing BSE makes them apprehensive (Table 4).

### 3.5. Determinants of Knowledge of BSE

A bivariate analysis was conducted to assess an association between each predictor variable and the outcome variable. The association of socio-demographic factors, personal and family history-related factors, and attitude-related factors with BSE knowledge was investigated. All variables that were associated with the outcome variable in the bivariate logistic regression at p <0.2 were included in the multivariate logistic regression model. After controlling the possible confounding variables, the last place of residence, attitude towards BSE, and knowing someone who had breast cancer were significantly associated with knowledge of BSE. Previously, urban residents were about two times more likely to have good knowledge of BSE than their rural counterparts (AOR =2.16, 95% CI (1.18–39.91), p =0.011). The odds of having good BSE knowledge were about three times more likely among those who had a good attitude than those who had a poor attitude (AOR =3.17, 95% CI (2.02–4.74), p <0.001). Those who knew someone with a diagnosis of breast cancer were almost three times more likely to have good knowledge than those who did not know. (AOR =2.95, 95% CI (1.77-4.91), p <0.001)**(**Table 5).

## 4. Discussion

### 4.1. Knowledge of BSE

For years, Ethiopia's health care system had overlooked the burden of non-communicable diseases. Cervical cancer and BC are the most common types of cancer in the nation, accounting for a sizable portion of the impact. The government has recently directed various efforts to improve the prevention, early detection, diagnosis, and management of these diseases [29].

BSE remains the most simple and cost-effective BC screening method for resource-constrained countries such as Ethiopia. If commenced appropriately at the right age, the procedure would allow early diagnosis and improve the survival of patients. This study sought to assess BSE knowledge and associated factors among female undergraduate students enrolled at Addis Ababa University. Almost half of the respondents were found to have good knowledge. BSE knowledge was significantly associated with attitude toward BSE, place of residence, and knowing someone who had BC.

The current study showed that 49.9% of the respondents had good BSE knowledge, which is lower compared to female medical students in Haramaya, University Ethiopia, where about 95% of the participants had good knowledge [26]. Another study from Gaza Strip-Palestine showed that most (94.1%) of the respondents correctly defined BSE [30]. The findings from the two studies are considerably higher than the present study due to the difference in the study population, which included medical students and health professionals, thus resulting in better awareness of the concept of BSE and exposure to patients diagnosed with BC. In contrast, lower percentages of BSE knowledge were reported in local institutional studies conducted in Adama, Hawassa, Gondar, and Debre Birhan, where only 8.7%, 23%, 27.6%, and 30.25% of female university students had good BSE knowledge, respectively [6, 11, 20, 31]. The relatively higher results in the present study compared to these local studies could be indicated by the area's relative urban nature. Urbanization consequently allows for better access to information regarding BSE.

Studies from Africa have shown higher BSE knowledge among higher education students. A survey from a Ugandan university showed that 76.5% of the participants had good BSE knowledge [9]. Another finding from Cameroon revealed that 73.5% of the students had heard of BSE, but about 62% were partially or substantially aware of BSE procedures [19]. The higher magnitudes from the two African countries may be attributed to variations in socio-economic conditions and definitions of BSE knowledge.

This study revealed that most of the participants ever heard of BSE. Another survey from Haramaya University, Ethiopia, showed that about 95.23% of the participants ever heard about BSE [26]. Even though the present study was conducted in a facility located in a more urban area, a comparable result was observed due to the latter study involving female medical students. In contrast to the two local studies, less than half (41.7%) of students from Gondar University, Ethiopia, reported that they had previously heard of BSE [11]. The results appear to differ due to variations in the study area and study population. Furthermore, the Gondar survey included summer social science students whose permanent residence is far from the city. This could have potentially limited their ability to obtain information about BSE.

Mass media has been cited as a highly efficient tool in enhancing health literacy. The dissemination of health information via radio and television has been shown to be an empowering tool for improving individual and public health [32]. Regarding creating awareness of BC and BSE, the mass media has played a great role. This is evident in the current study, as 64.3% of respondents cited the media as their primary source of information regarding BSE. Two studies from Africa also reported similar findings. A study from Uganda revealed that most (56.9%) of the female university students mentioned the mass media as their source of information about BSE [9]. A Cameroonian study also indicated that the media is one of the factors that influenced students to be aware of and perform BSE [19].

According to this study, 25% of the respondents mentioned that BSE should be performed every month, while 60 (14.7%) of the respondents indicated that BSE should be performed after menstruation. On the other hand, a study from the State of Palestine reported that almost 70% of the participants knew the right time to conduct BSE, which is a few days after menstruation [33]. About 56% of students in this study acknowledged BSE as a form of BC screening. The majority (82%) of the students in a Jordanian university stated that BSE is an effective method in terms of screening BC [34]. Comparatively, the results of the international studies identified a better understanding of BSE due to the participants' being health science students whose syllabuses include topics regarding women's health, including BSE.

The findings of this study showed that among the respondents who had ever heard of BSE, more than half (52.6%) of them reported that BSE enables the detection of atypical changes in the size and form of the breasts. This same group of respondents also indicated that BSE allows for the understanding of how the breasts ordinarily feel and seem. Additionally, about 131 (32%) respondents correctly identified the proper age to begin BSE as after 19 years of age. Consistent with this finding is a study from Debre Birhan, Ethiopia, where 30.5% of female students knew when to perform BSE [6] A large proportion (77.7%) of undergraduate students in Pakistan also identified the appropriate age to start BSE [14]. The latter study's finding is higher due to the significant socioeconomic difference between the two countries. This would lead to a difference in the amount of money spent on health care to deal with the burden of BC, which would include more efforts to raise awareness of BSE.

### 4.2. Determinants of BSE Knowledge

The present study elucidated that respondents who knew somebody who had BC were found to have good knowledge than their counterparts (AOR =2.95, 95% CI (1.77-4.91), p <0.001). In line with this study, a Saudi study found that knowing someone who has BC is a significant predictor of having good knowledge of BSE [35]. Furthermore, these findings were supported by a study from Brazil, which found that knowing someone with BC had a significant influence on adherence to BSE [36]. These similarities across the different studies stem from an increased interest in understanding BSE as a result of the experience with the severity of BC.

Access to health information, such as the benefits of BSE, is generally thought to be better among people living in the cities. In Ethiopia, this is due, in part, to socioeconomic differences between urban and rural dwellers, which create a disparity in terms of access to mass media. For example, a girl who has spent the majority of her life in areas far from relatively developed cities may only have one radio per household, which under most circumstances is inaccessible to her. Furthermore, the number of health institutions that can be used as a setting for health education, as well as the availability of skilled health care providers who can disseminate information, is limited in rural cities. According to this study, the odds of having good knowledge were about two times higher among participants who lived in an urban area before joining the university than among those who previously lived in a rural area (AOR =2.16, 95% CI (1.19–39.92), p = 0.011).

Respondents who had a good attitude towards BSE were found to have better knowledge than those who had a poor attitude (AOR = 3.17, 95% CI (2.02–4.74), p <0.001). The finding may indicate that having a favorable attitude towards the BSE process may influence women to further read and understand the concept and ultimately perform the procedure.

### 4.3. Limitations

The scarcity of research using similar measurement parameters has made comparisons difficult. The cross-sectional design is limited in showing the direction of the relationship between exposure and outcome factors. In addition, a qualitative study that could have added to the quantitative data was not done.

## 5. Conclusion and Recommendation

According to the findings of this study, less than half of the participants had a thorough understanding of BSE. Short-term breast self-examination training should be organized to close the knowledge gap among college/university students. To raise awareness of breast cancer, educational campaigns and the formation of peer groups at the university level should be facilitated, with a focus on early detection measures. The media should also provide adequate coverage to raise awareness about BSE.

## Figures and Tables

**Table 1:** Socio-demographic characteristics of female students at CoBE, Addis Ababa University, Ethiopia (n = 407).

Variables	Frequency (407)	Percentage (%)
*Age*		
20-22	347	85.3
23 and above	60	14.7
*Marital status*		
Single	387	95.1
Married	16	3.9
Divorced/separated	4	1.0
*Previous place of residence*		
Urban	342	84.0
Rural	65	16.0
*Year of study*		
1^st^ year	109	26.8
2^nd^ year	170	41.8
3^rd^ year	128	31.4
*Family average monthly income in ETB($)*		
<445 (<20$)	12	2.9
446-1200(21-55$)	27	6.7
1201-2500(56-114$)	79	19.4
2501-3500(115-160$)	39	9.6
>3501(>161$)	250	61.4
*Family education status*		
Illiterate	23	5.7
Read and write	45	11.0
Elementary school	27	6.6
Secondary school and above	312	76.7
*Ethnicity*		
Amhara	182	44.7
Oromo	96	23.6
Tigre	61	15.0
Gurage	61	15.0
Others∗	7	1.7

Others∗ Silte, Sidama, Hadiya, ETB – Ethiopian Birr, $ - United States Dollars.

**Table 2:** Personal and family history of female students at CoBE, Addis Ababa University, Ethiopia (n = 407).

Variables	Frequency (407)	Percentage (%)
*Family history of BC*		
Yes	41	10.1
No	366	89.9
*Family member who had a history of breast cancer (n =41)*		
Mother	7	17.1
Sister	1	2.4
Grandmother	12	29.3
Aunt	21	51.2
*Personal history of benign breast problems*		
Yes	13	3.2
No	394	96.8
*Knew someone with BC*		
Yes	99	24.3
No	308	75.7

**Table 3:** Knowledge of BSE among female students at CoBE, Addis Ababa University, Ethiopia.

Variables	Frequency	Percent (%)
*Ever heard of BSE*		
Yes	376	92.4
No	31	7.6
*Had received extended information on BSE*		
Yes	207	50.9
No	200	49.1
*Source of information for BSE*∗*n =221*		
Health care provider	34	15.4
Colleagues/friends	40	18.1
Mass media	142	64.2
Others	5	2.3
*Time to begin performing BSE*		
≤19 years	53	13.0
>19 years	131	32.2
I do not know	223	54.8
*The appropriate frequency of BSE practice*		
Every week	44	10.8
Every month	101	24.8
Every year	36	8.8
I do not know	226	55.6
*The appropriate time to perform BSE*		
Before the period starts	29	7.1
After the period starts	60	14.7
Any time of the month	57	14.0
I do not know	261	64.2
*Early detection of BC improves the chance of survival*		
Yes	282	69.3
No	102	25.0
I do not know	23	5.7
*Awareness of other types of BC screening methods*		
CBE	229	56.2
Mammography	63	15.5
I do not know	115	28.3
*BSE enables the detection of atypical changes in the size and form of the breasts and understanding of how the breasts ordinarily feel and seem. (n =376)*		
Yes	198	52.6
No	178	47.4
*BSE should be done in front of the mirror (n =376)*		
Yes	224	59.6
No	152	40.4

∗Total number is not equal to ‘n' due to multiple responses.

**Table 4:** Frequency distribution of attitude towards BSE among female students in Addis Ababa University, Ethiopia.

Statement	Strongly disagree (%)	Disagree (%)	Neutral (%)	Agree (%)	Strongly agree (%)
Performing BSE makes me feel so absurd.	50 (12.3)	98(24.1)	165(40.5)	73(17.9)	21(5.2)
I am motivated to perform BSE.	7(1.7)	25(6.1)	119(29.2)	189(46.5)	67(16.5)
BSE is a subject that I frequently discuss with my friends.	12(3.0)	50(12.3)	125(30.7)	167(41.0)	53(13.0)
I am constantly on the lookout for information regarding BSE.	47(11.5)	94(23.1)	168(41.3)	74(18.2)	24(5.9)
Conducting BSE is a waste of time.	159(39.1)	159(39.1)	61(14.9)	24(5.9)	4(1.0)
I hesitate to perform BSE as it is awkward	12(3.0)	25(6.1)	98(24.1)	142(34.9)	130(31.9)
I avoid BSE for fear of contracting breast cancer.	15(3.7)	52(12.8)	80(19.6)	138(33.9)	122(30.0)
I'm apprehensive about BSE.	114(28.0)	127(31.2)	101(24.8)	50(12.3)	15(3.7)

**Table 5:** Factors associated with knowledge of BSE among female students in Addis Ababa University, Ethiopia.

Variables	Knowledge of BSE		COR (95% CI)	AOR(95% CI)	p-value
	Poor knowledge n (%)	Good knowledge n (%)			
*Previous place of residence*					
Urban	75(21.9)	267(78.1)	0.81(1.21-1.59)	**2.16(1.19-3.92)**∗	**0.011**∗
Rural	12(18.5)	53(81.5)	1.00	**1.00**	
*Family average monthly income*					
<445 (<20$)	7(58.3)	5(41.7)	0.58(0.18-1.88)	0.74(0.20-2.70)	0.648
446-1200(21-55$)	17(63.0)	10(37.0)	0.48(0.21-1.08)	0.74(0.30-1.86)	0.528
1201-2500(56-114$)	40(50.6)	39(49.4)	0.79(0.48-1.31)	0.96(0.54-1.70)	0.891
2501-3500(115-160$)	28(71.8)	11(28.2)	0.32(0.15-0.67)	0.57(0.25-1.30)	0.176
>3501(>161$)	112(44.8)	138(55.2)	1.00	1.00	
*Attitude towards BSE*					
Good attitude	74(35.9)	132(64.1)	3.26(2.18-4.90)	**3.17(2.02-4.74)**∗	**<0.001**∗
Poor attitude	130(64.7)	71(35.3)	1.00	**1.00**	
*Family educational status*					
Illiterate	15(65.2)	8(34.8)	0.47(0.19-1.14)	0.59(0.23-1.54)	0.284
Read and write	24(53.3)	21(46.7)	0.77(0.41-1.44)	1.27(0.62-2.60)	
Elementary					
School	19(70.4)	8(29.6)	0.37(0.16-0.87)	0.52(0.21-1.30)	0.516
Secondary school					
And above	146(46.8)	166(53.2)	1.00	1.00	0.159
*Knew someone who had BC*					
Yes	30(30.3)	69(69.7)	2.99(1.84-4.85)	**2.95(1.77-4.91)**∗	**<0.001**∗
No	174(56.5)	134(43.5)	1.00	**1.00**	

∗Statistically significant at p-value <0.05.

## Data Availability

The datasets used and/or analyzed during the current study are available from the corresponding author on reasonable request.

## Abbreviations

AOR: Adjusted odds ratio
BC: Breast Cancer
BSE: Breast self-examination
CBE: Clinical breast examination
CI: Confidence interval
COR: Crude odds ratio
ETB: Ethiopian birr
SPSS: Statistical package for social science
USD: United States Dollar.

## Supplementary Materials

Supplementary Materials STROBE Statement—Checklist of items that should be included in reports of *cross-sectional studies.*

## References

[1] Sung H., Ferlay J., Siegel R. L. (2021). Global Cancer Statistics 2020: GLOBOCAN Estimates of Incidence and Mortality Worldwide for 36 Cancers in 185 Countries. *A Cancer Journal for Clinicians*.

[2] Azubuike S. O., Muirhead C., Hayes L., McNally R. (2018). Rising global burden of breast cancer: the case of sub-Saharan Africa (with emphasis on Nigeria) and implications for regional development: a review. *World Journal of Surgical Oncology*.

[3] Gebremariam A., Addissie A., Worku A. (2019). Breast and cervical cancer patients' experience in Addis Ababa city, Ethiopia: a follow-up study protocol. *BMJ Open*.

[4] Hadgu E., Seifu D., Tigneh W. (2018). Breast cancer in Ethiopia: evidence for geographic difference in the distribution of molecular subtypes in Africa. *BMC Women's Health*.

[5] Tesfaw A., Alebachew W., Tiruneh M. (2020). Why women with breast cancer presented late to health care facility in north-West Ethiopia? A qualitative study. *PLoS One*.

[6] Birhane K., Alemayehu M., Anawte B. (2017). Practices of breast self-examination and associated factors among female Debre Berhan University students. *International Journal of Breast Cancer*.

[7] Fondjo L. A., Owusu-Afriyie O., Sakyi S. A. (2018). Comparative Assessment of Knowledge, Attitudes, and Practice of Breast Self- Examination among Female Secondary and Tertiary School Students in Ghana. *International Journal of Breast Cancer*.

[8] Gebresillassie B. M., Gebreyohannes E. A., Belachew S. A., Emiru Y. K. (2018). Evaluation of knowledge, perception, and risk awareness about breast Cancer and its treatment outcome Among University of Gondar Students, Northwest Ethiopia. *Frontiers in Oncology*.

[9] Godfrey K., Agatha T., Nankumbi J. (2016). Breast Cancer knowledge and breast self-examination practices among Female University students in Kampala, Uganda: a descriptive study. *Oman Medical Journal*.

[10] Ibitoye O. F., Thupayegale-Tshwenegae G. (2021). The impact of education on knowledge attitude and practice of breast self-examination among adolescents girls at the Fiwasaye girls grammar school Akure, Nigeria. *Journal of Cancer Education*.

[11] Mihret M. S., Gudayu T. W., Abebe A. S. (2021). Knowledge and Practice on Breast Self-Examination and Associated Factors among Summer Class Social Science Undergraduate Female Students in the University of Gondar, Northwest Ethiopia. *Journal of Cancer Epidemiology*.

[12] Omar A., Bakr A., Ibrahim N. (2020). Female medical students' awareness, attitudes, and knowledge about early detection of breast cancer in Syrian Private University, Syria. *Heliyon.*.

[13] Lera T., Beyene A., Bekele B., Abreha S. (2020). Breast self-examination and associated factors among women in Wolaita Sodo, Ethiopia: a community-based cross-sectional study. *BMC Women's Health*.

[14] Hussain I., Majeed A., Masood I. (2022). A national survey to assess breast cancer awareness among the female university students of Pakistan. *PLoS One*.

[15] Madhukumar S., Thambiran U. R., Basavaraju B., Bedadala M. R. (2017). A study on awareness about breast carcinoma and practice of breast self-examination among basic sciences' college students, Bengaluru. *Journal of Family Mdicine and Primary care*.

[16] Akhtari-Zavare M., Juni M. H., Ismail I. Z., Said S. M., Latiff L. A. (2015). Barriers to breast self examination practice among Malaysian female students: a cross sectional study. *Springerplus*.

[17] Rahman S. A., Al-Marzouki A., Otim M., Khalil Khayat N. E. H., Yousuf R., Rahman P. (2019). Awareness about breast Cancer and breast self-examination among female students at the University of Sharjah: a cross-sectional study. *Asian Pacific Journal of Cancer Prevention*.

[18] Ossai E. N., Azuogu B. N., Ogaranya I. O., Ogenyi A. I., Enemor D. O., Nwafor M. A. (2019). Predictors of practice of breast self-examination: a study among female undergraduates of Ebonyi State University, Abakaliki, Nigeria. *Nigerian Journal of Clinical Practice*.

[19] Nde F. P., Assob J. C., Kwenti T. E., Njunda A. L., Tainenbe T. R. (2015). Knowledge, attitude and practice of breast self-examination among female undergraduate students in the University of Buea. *BMC Research Notes*.

[20] Tafa Segni M., Tadesse D. M. (2015). Breast Self-examination: Knowledge, attitude, and practice among female health science students at Adama science and Technology University, Ethiopia. *Gynecology & Obstetrics*.

[21] Yohannes Mehretie Adinew A. G. W., Mengesha Z. B. (2013). Knowledge of reproductive and sexual rights among university students in Ethiopia: institution-based cross-sectional. *BMC International Health & Human Rights*.

[22] (2016). *Registrar office of the CoBE*.

[23] Hailu T. (2014). Knowledge of breast Cancer and its early detection measures among female students, in Mekelle University, Tigray region, Ethiopia. *Clinical Medicine*.

[24] Suh M. A. B., Atashili J., Fuh E. A., Eta V. A. (2012). Breast Self-Examination and breast cancer awareness in women in developing countries: a survey of women in Buea, Cameroon. *BMC Res Notes*.

[25] Lemlem S. B., Sinishaw W., Hailu M., Abebe M., Aregay A. (2013). Assessment of knowledge of breast Cancer and screening methods among nurses in university hospitals in Addis Ababa, Ethiopia, 2011. *International Scholarly Research Notices*.

[26] Kalandar Ameer S. M. A., Pal S. K., Arebo K., Kassa G. G. (2014). Breast Cancer awareness and practice of breast self-examination among female medical. International journal of interdisciplinary and multidisciplinary. *Studies*.

[27] Makanjuola O. J. A. P. O., Ajibade B. L., Makinde O. Y., Cancer B. (2013). Breast Cancer: Knowledge and practice of breast self examination among women in rural community of Ondo state, Nigeria. *Journal of Pharmacy and Biological Sciences*.

[28] Lavdaniti M. (2015). Perceptions and health beliefs of Greek nursing students about breast self- examination: A descriptive study. *International Journal of Nursing Practice*.

[29] Solomon S., Mulugeta W. (2019). Diagnosis and risk factors of advantage cancers in Ethiopia. *Journal of Cancer Prevention*.

[30] Mansour H. H., Shallouf F. A., Najim A. A., Alajerami Y. S., Abushab K. M. (2021). Knowledge and practices of female nurses at primary health care clinics in Gaza strip-Palestine regarding early detection of breast Cancer. *Asian Pacific Journal of Cancer Prevention*.

[31] Abera H., Mengistu D., Bedaso A. (2017). Effectiveness of planned teaching intervention on knowledge and practice of breast self-examination among first year midwifery students. *PLoS One*.

[32] Levin-Zamir D., Bertschi I. (2018). Media health literacy, eHealth literacy, and the role of the social environment in context. *International Journal of Environmental Research and Public Health*.

[33] Abo Al-Shiekh S. S., Ibrahim M. A., Alajerami Y. S. (2021). Breast Cancer Knowledge and Practice of Breast Self-Examination among Female University Students, Gaza. *Scientific World Journal*.

[34] Abu Sharour L., Al-Ghabeesh S., Suleiman K., Salameh A. B., Jacoob S., Al-Kalaldeh M. (2017). Predictors of breast self-examination performance among Jordanian university female students. *Eur J Cancer Care (Engl).*.

[35] Latif R. (2014). Knowledge and attitude of Saudi female students towards breast cancer: a cross-sectional study. *Journal of Taibah University Medical Sciences.*.

[36] Brum I. V., Rodrigues T., Laporte E. G. J., Aarestrup F. M., Vitral G. S. F., Laporte B. E. P. (2018). Does knowing someone with breast Cancer influence the prevalence of adherence to breast and cervical Cancer screening?. *Revista Brasileira de Ginecologia e Obstetrícia*.

